# Quorum sensing in the probiotic bacterium *Escherichia coli* Nissle 1917 (Mutaflor) – evidence that furanosyl borate diester (AI-2) is influencing the cytokine expression in the DSS colitis mouse model

**DOI:** 10.1186/1757-4749-4-8

**Published:** 2012-08-03

**Authors:** Christoph A Jacobi, Stefanie Grundler, Chih-Jen Hsieh, Julia Stefanie Frick, Patrick Adam, Georg Lamprecht, Ingo B Autenrieth, Michael Gregor, Peter Malfertheiner

**Affiliations:** 1 Department of Internal Medicine I, University clinic Tübingen, Otfried Müllerstr. 10, 72076, Tübingen, Germany; 2 Institute of Medical Microbiology and Hygiene, University clinic Tübingen, Otfried Müllerstr. 10, 72076, Tübingen, Germany; 3 Institute for Pathology and Neuropathology, University clinic Tübingen, Otfried Müllerstr. 10, 72076, Tübingen, Germany; 4 Department for Gastroenterology, Hepatology and Infectiology, University clinic Magdeburg, Leipzigerstr. 44, 39120, Magdeburg, Germany

**Keywords:** Quorum sensing, *Escherichia coli* Nissle, Autoinducer-2, DSS colitis, Cytokines

## Abstract

**Background:**

“Quorum sensing” (QS) is the phenomenon which allows single bacterial cells to measure the concentration of bacterial signal molecules. Two principle different QS systems are known, the Autoinducer 1 system (AI-1) for the intraspecies communication using different Acyl-homoserine lactones (AHL) and AI-2 for the interspecies communication. Aim of this study was to investigate QS of *Escherichia coli* Nissle 1917 (Mutaflor).

**Results:**

While *E. coli* Nissle is producing AI-2 in a density dependent manner, no AI-1 was produced. To study the effect of AI-2 in the DSS (dextran sulphate sodium) induced mouse model of acute colitis, we silenced the corresponding gene *lux*S by intron insertion. The mutant bacterium *E. coli* Nissle::*lux*S was equally effective in colonizing the colon and the mutation turned out to be 100% stable during the course of the experiment. Isolating RNA from the colon mucosa and performing semiquantitative RT PCR, we were able to show that the expression of the pro-inflammatory cytokine IFN-y was suppressed in mice being infected with the *E. coli* Nissle wild type. Mice infected with the *E. coli* Nissle::*lux*S mutant showed a suppressed expression of IL-10 compared to uninfected mice, while the expression of the pro-inflammatory cytokines IL-6 and TNF-α was higher in these mice. The expression of mBD-1 was suppressed in mice being infected with the mutant in comparison to the mice not infected or infected with the wild type. No differences were seen in the histological examination of the colon sections in the different groups of mice.

**Conclusions:**

*E. coli* Nissle is producing AI-2 molecules, which are influencing the expression of cytokines in the mucosa of the colon in the DSS mice. However, if QS has a direct influence on the probiotic properties of *E. coli* Nissle remains to be elucidated.

## Background

The communication of bacteria with each other is termed “quorum sensing” (QS) [1]. It is an important global gene regulatory mechanism, which is used by gram-negative as well as gram-positive bacteria, enabling individual bacteria to communicate and coordinate their behavior in populations. In general terms, it is often defined as cell density-dependent regulation of gene expression via extracellular signals. Bacteria produce small, diffusible signals, termed “autoinducers”. When these signals reach a critical threshold concentration, the targeted QS genes are activated or repressed. Acyl-homoserine lactone signal-mediated QS systems are the primary QS system discovered in gram-negative bacteria. AHL were originally identified in marine bacteria, where they play a pivotal role in the regulation of bioluminescence in *Vibrio fischeri*[2-4]*.* Bacteria may produce one or more different AHL, which regulate diverse phenotypes, such as biofilm formation, swarming, production of proteases, antibiotics, siderophores or bioluminescence, conjugation, the modulation of the immune system and the induction of apoptosis [5-11]. While AHL are responsible for intraspecies communication, a second Autoinducer (AI-2) was discovered, which is responsible for the interspecies communication; furanosyl borate diester is synthezised by the gene *lux*S, which is found in the genome of many gram-negative as well as gram-positive bacteria. It is an integral component of the activated methyl cycle, which detoxifies S-adenosyl-L-Methionine (SAM) [12-14].

Probiotics are, according to the FAO/WHO “live bacteria which when administered in adequate amounts confer to a health benefit to the host”. The numerous mechanisms of the probiotic acting microorganisms include induced expression of certain cytokines, as well as increased secretion of immunoglobulin A and mucin, activation of lymphocytes and macrophages and inhibition of the adhesion and invasion of epithelial cells [15]. *Escherichia coli* Nissle 1917 (Mutaflor) is one of the most extensively studied probiotic bacterium. It was isolated in 1917 by Prof. Nissle from feces from a soldier during the First World War, who did not suffer from diarrhoea as his comrades did. Prof. Nissle realized the potential health benefits early on [16]. In addition, several important discoveries were made during recent years: *E. coli* Nissle is inducing human ß-defensin 2 (hBD-2) expression in the cell culture in a time and density dependent manner [17]. In another study it has been shown that *E. coli* Nissle is decreasing TNF-α secretion [18]. Also, *E. coli* Nissle is outcompeting several nonpathogenic and pathogenic bacteria including ETEC and EPEC strains in biofilm formation [19]. Maintaining remission in ulcerative colitis using *E. coli* Nissle have shown equivalent efficacy to the gold standard mesalazine [20-22]. However, the exact mechanisms by which *E. coli* Nissle is exerting its beneficial effects are still not completely understood [23].

If *E. coli* Nissle is using QS to communicate with itself or with other species has not been elucidate so far. In this report we want to elucidate whether or not this bacterium is using QS. For the first time we were able to show that *E. coli* Nissle is producing AI-2 in a density dependent manner. We silenced the *lux*S gene by intron insertion and studied the effect of QS in the DSS mouse model of acute colitis. We observed differential expression of different cytokines and mBD-1, suggesting that indeed QS is influencing the probiotic properties of *E. coli* Nissle.

## Materials and methods

### Animals

Female C57BL/6 J (B6) mice (6–8 weeks of age) were purchased from Harlan Winkelmann (Borchen, Germany) and were kept under SPF conditions. The animals were handled in strict accordance with good animal practice and all animal work was approved by an appropriate institutional review committee (Anzeige vom 01.05.2006 Regierungspräsidium Tübingen).

### Bacterial strains and growth conditions

The *Escherichia coli* strain Nissle 1917 (Mutaflor) was kindly provided to us by Dr. Sonnenborn from Ardeypharm (Herdecke, Germany). The *lux*S mutant of *E. coli* Nissle 1917 (*E. coli* Nissle::*lux*S) was generated by intron II insertion using the Targetron System (Sigma Aldrich, Munich, Germany). Using the sequence of *lux*S, PCR primers (Table 1.) were generated to mutate the intron. The mutated intron was cloned into the supplied vector pACD4K, which was transferred into *E. coli* Nissle. The “target site”(insertion site for the intron) of the *lux*S gene was determined to be the following sequence: CGATATCTCGCCAATGGGCTGCCGCACCGG – intron – TTTTTATATGAGTCT. The correct insertion of the intron in the *lux*S gene was confirmed by PCR (see Additional file 1). In addition, experiments were conducted showing that AI-2 was not produced any longer by its mutant. The strains were routinely grown in Luria-Bertani (LB) broth at 37°C, kanamycin (50 μg/ml) was added when growing the mutant.

**Table 1:** Bacterial strains and primers used

**Name**	**Sequence (5'→3')**
Primer for *E.coli Nissle Mutaflor*	
Mut7f	GACCAAGCGATAACCGGATG
Mut8r	GTGAGATGATGGCCACGATT
Primer for “Targetron”mutation:	
IBS	AAAAAAGCTTATAATTATCCTTACTGCCCCACCGG
	GTGCGCCCAGATAGGGTG
EBS1d	CAGATTGTACAAATGTGGTGATAACAGATAAGTCC
	ACCGGTTTAACTTACCTTTCTTTGT
EBS2	TGAACGCAAGTTTCTAATTTCGGTTGGCAGTCGAT
	AGAGGAAAGTGTCT
EBSuniv	CGAAATTAGAAACTTGCGTTCAGTAAAC
RT PCR Primer:	
GAPDHf	CCAGCCGAGCCACATCGCTC
GAPDHr	ATGAGCCCCAGCCTTCTCCAT
IFN-γf	TCAAGTGGCATAGATGTGGAAGAA
IFN-γr	TGGCTCTTGCAGGATTTTCATG
IL-6f	GAGGATACCACTCCCAACAGACC
IL-6r	AAGTGCATCATCGTTGTTCATACA
IL-10f	AGGCGCTGTCATCGATTTCTC
IL-10r	TGGCCTTGTAGACACCTTGGTC
mBD-1f	ATGAAAACTCATTACTTTCTCCTGG
mBD-1r	ACTACTGTCAGCTCTTACAACA
TNF-αf	GCAAGCTTCGCTCTTCTGTCTACTGAACTTCGG
TNF-αr	GCTCTAGAATGAGATAGCAAATCGGCTGACGG
Bacteria:	
DH5α [pSB403]	DH5α carrying plasmid pSB403
*E. coli* Nissle 1917	DSM6601; Serovar 06:K5:H1
*E. coli* Nissle::*lux*S	Nissle mutated in the *lux*S gene
*Vibrio harveyi* BB*120*	*Vibrio harveyi* wildtype
*Vibrio harveyi* BB*886*	*Vibrio harveyi* AI-1 Sensor
*Vibrio harveyi* BB*170*	*Vibrio harveyi* AI-2 Sensor

For the mouse experiments, the bacteria were grown to an OD_600_ = 1 (~0.8–1.2 × 10^9^ CFU/ml) from an overnight culture diluted 1:100 in LB broth. The bacteria were collected by centrifugation, washed once in PBS and were resuspended in 200 ml drinking water (+ 4% DSS in the course of the experiment) for the mice.

### Detection of QS molecules

We attempted to isolate AI-1 molecules as it was described before [24]. Extraction of culture supernatant (50 ml, corresponding to 5 × 10^10^ bacteria) yielded 500 μl of AHL concentrate in ethyl acetate. Appropriate dilutions of the concentrates were loaded on a thin-layer chromatography (TLC) plate (RP-18 F_254S_; 20 by 20 cm; Merck, Darmstadt, Germany) and were run in a moisture chamber containing a mixture of 60% methanol and 40% distilled water for 6 h [25]. After the plate was dried, it was overlaid with 200 ml of 0.6% soft LB agar seeded with 20 ml of a logarithmically grown culture of the *lux*-based AHL biosensor strain *E*. *coli* [pSB403], which is able to respond to a range of different AHL by luciferase production (e.g., BHL, HHL, OHHL, and ODHL) [26]. The incubation was carried out overnight in a moisture chamber at 30°C, and AHLs were detected via autoradiography.

Autoinducer-2 (AI-2) levels in cell-free culture supernatant were measured using the *Vibrio harveyi* bioluminescence assay as described previously [13]. Briefly, the bacteria were grown as described above and cell-free supernatant was prepared at different time points by centrifugation of the sample at 10.000 × *g* for 10 minutes. The supernatant was immediately frozen at −80°C until being subjected to AI measurements: a 60 μL aliquot of each sample was added to 600 μl of the sensor bacterium *V. harveyi* BB170 (AI-2) or *V. harveyi* BB886 (AI-1) (being diluted 1:5.000 after being grown in “AB” medium) [13] and was incubated at 30°C. Every hour the bioluminescence was measured using a Wallac Luminometer (Freiburg, Germany). All measurements were reported at the three hour incubation period, when the difference between negative controls and positive controls reached maximal levels. The measurements with the DH5α [pSB403] sensor bacterium was performed in a similar fashion, however the luminescence was measured after overnight incubation.

### Experiments with DSS colitis mice

**Figure 1:**
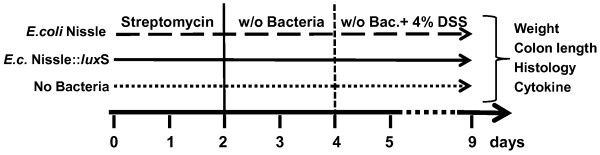
** Experimental design of the DSS colitis model.** The bacterial decontamination of the mouse intestine was performed by streptomycin treatment for the first two days of the experiment, followed by oral feeding with *E. coli* Nissle, *E. coli* Nissle::*lux*S or pure drinking water. Acute colitis was induced by administration of 4% DSS in addition to the bacteria to the drinking water, starting from day five of the experiment.

After bacterial decontamination of the intestine with streptomycin (5 μg/ml in drinking water) for two days [27], the success of this procedure was monitored by plating out stool on blood agar, LB agar and McConkey Agar and incubating them for three days under aerobic and anaerobic conditions at 37°C. From day three the bacteria were administered in the drinking water of the mice, which was replaced every two days. Starting at day five of the experiment, 4% of DSS was dissolved in the drinking water to induce acute colitis (Figure 1). The viability of the bacteria was not affected by the presence of the DSS, as was checked by plating the suspension on agar plates. The weight of each mouse was recorded every day and the whole experiment ended at day nine when the maximal loss of weight was 15% of their initial weight. The mice were killed in a CO_2_ containing atmosphere. The large intestine was removed under sterile conditions. The length of the colon was measured. Stool was weighted, resuspended, homogenized, diluted and plated on blood-, LB- and McConkey plates and were incubated in an anaerobic and aerobic atmosphere at 37°C. To confirm that the isolated bacteria where indeed *E. coli* Nissle wild type or mutant, we performed colony PCR with Nissle specific primers (Table 1). For this we extracted bacterial DNA by suspending bacterial cell mass in steril water and incubating the suspension at 95°C for 10 minutes. After centrifugation (5.000 rpm; 5 minutes), the DNA containing supernatant was used for PCR [28]. The mouse experiments were repeated twice, each group of mice consisted of three to five mice. 

### Expression of cytokines and mouse beta-defensin-1 from mucosa of the colon

To analyze the cytokine and mouse beta defensin-1 (mBD-1) mRNA expression in the colonic mucosa, the mucosa (about 0.5 cm^2^) was carefully scraped off the colon. RNA was isolated using the RNAeasy Minikit (Qiagen, Hilden, Germany). Isolated RNA was reverse transcribed with Superscript reverse transcriptase (Invitrogen, Karlsruhe, Germany), oligodT- and random hexamer primers (Invitrogen). RT PCR was performed with the primers denoted in Table 1 using 25 ng of cDNA template on an ABI Prism 7000 System using the SYBR green method (Fermentas, St. Leon Rot, Germany). Thermal cycling conditions were: 95°C/10 min followed by 40 cycles of 92°C/15 s and 60°C/60 s. Detection of fluorescent signal was performed according to the recommended protocols for the ABI Prism 7000 Real Time PCR machine (Applied Biosystems, Foster City, California, USA) The data was analyzed by the ΔC_T_ method using GAPDH for normalization (ΔC_T_ = C_T_^sample^ - C_T_^GAPDH^). For each increase in ΔC_T_ (x), the expression is increased by a factor of 2^x^[29].

### Histological scoring

Colon tissue was fixed in ice-cold neutral buffered 4% formalin for at least two hours followed by a washing step in phosphate buffered saline. For cryoprotection the tissue was treated in a 30% saccharose solution overnight. Afterwards, the tissue was embedded in paraffin and cut into 2 μm sections. Samples were stained with hematoxylin and eosin (H&E) (Merck, Darmstadt, Germany). Sections were analyzed in a blinded fashion by one pathologist.

### Statistical analysis

All data are presented as means ± S.D. Analysis of variance and students *t* tests were applied when appropriate.

## Results

### AI-1/AI-2 production in *E. coli* Nissle 1917

**Figure 2:**
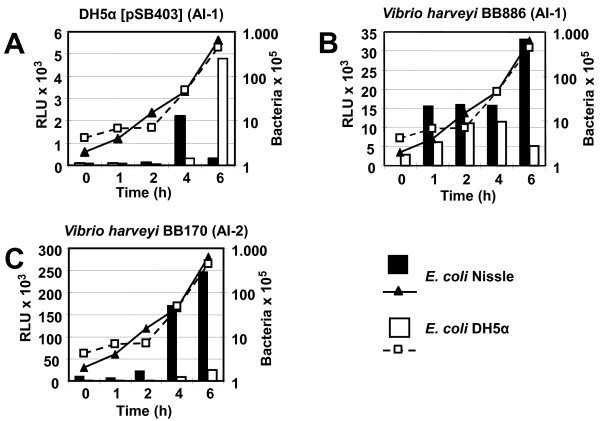
** AI-1 and AI-2 production in *E.coli* Nissle and *E.coli* DH5α with the sensor bacteria *E.coli* DH5α [pSB403] (A), *V. harveyi* BB886 (B) and *V. harveyi* BB170 (C).** Overnight cultures of *E.coli* Nissle and *E.coli* DH5α were diluted 1:100 in LB medium containing 0.5% glucose. At the given time intervals aliquots were drawn, the bacterial numbers were determined by serial dilutions (lines; right Y-axis). Bacterial supernatants were obtained by centrifugation and frozen at −80°C. The presence of AI-1 and AI-2 was determined by incubating the respective sensor bacteria with the supernatants (via the luminescence (bars) – left Y-axis).

To elucidate whether or not *E. coli* Nissle is communicating via QS molecules, we attempted to detect QS signal molecules. In case of AI-1, we tried to isolate and detect homoserine lactones. The direct isolation of AHL from the culture supernatant failed (data not shown), as did the indirect detection via two different sensor strains, DH5α [pSB403] and *Vibrio harveyi* BB886 (Figure 2A; B): while the number of bacteria were increasing during the growth phase as shown by the lines in the diagrams, the luminescence emitted by the sensor strains was low. Eventhough the luminescence was doubled after six hours of incubation, it still remained at background level. However, when we attempted to detect AI-2 from the supernatant of the bacterial cultures, we were successful. Employing the AI-2 sensor strain *Vibrio harveyi* BB170, we detected furanosyl borate diester in a density dependent manner: after four hours of incubation, the luminescence was more than six times the amount (in comparison to the two hours incubation period). And after six hours the luminescence was 10-fold higher. Thus, for the first time, we are able to show that *E. coli* Nissle is producing AI-2. In the logarithmic and late logarithmic phase of the growth curve, the production of AI-2 reached its maximum. Here the luminescence was more than 10-fold higher than the luminescence of the AI-1 sensor strain *Vibrio harveyi* BB886 (Figure 2C). During the later phases of growth, the AI-2 production is leveling off again (data not shown). Using specific primers derived from the *E. coli* K12 *lux*S sequence (Table 1), we were able to amplify the *lux*S gene from *E. coli* Nissle (data not shown).

In order to study the effect of AI-2 of *E. coli* Nissle in the DSS mouse model of acute colitis, we mutated the *lux*S gene by intron insertion. Genotypic and phenotypic control experiments confirmed the mutation: the intron II was inserted into the *lux*S gene. No AI-2 was detected in the supernatant of the mutant as compared to the corresponding wild type *E. coli* Nissle (see Additional file 1).

### Colonisation and stability of *E. coli* Nissle and *E. coli* Nissle::*lux*S in the DSS mouse model of acute colitis

**Figure 3:**
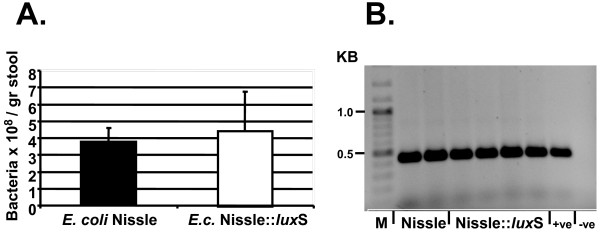
** Control experiments for colonization (A) and stability (B).** At the end of each experiment stool was weighted, homogenized, diluted and plated on agar followed by incubation at 37°C for three days. Bacterial colonies were counted **(A)**. Colony PCR with Nissle specific primers (Table 1) was performed on selected bacterial clones. On the left is the molecular weight standard in kilobases (KB) **(B).**

For the first time, we showed that *E. coli* Nissle is producing AI-2 molecules. In the next series of experiments, we elucidated whether or not AI-2 produced by *E. coli* Nissle has an influence in mice. For this, we used a DSS model of acute colitis in female C57/BL6 mice. In a first step, we performed control experiments to elucidate the effect of the *lux*S mutation in comparison to the wild type on the colonization and stability in mice. The bacteria were supplied in the drinking water of the mice (Figure 3). The rate of colonization of *E. coli* Nissle and its correspondent *E. coli* Nissle::*lux*S mutant were very similar as was shown by diluting and plating the stool on different agar plates (Figure 3A). To confirm that the isolated bacteria are indeed *E. coli* Nissle, a PCR with Nissle specific primers was performed. The isolated clones from the plates were either *E. coli* Nissle wild type or the *E. coli* Nissle::*lux*S mutant; the Nissle specific PCR was positive, as shown by the a specific amplified PCR band (Figure 3B). In another series of experiments we confirmed the stability of the mutant in the mice: agar plates with and without the antibiotic kanamycin were inoculated with clones from mice, which were fed with the mutant. 100% of the clones were resistant to kanamycin confirming the stability of the *lux*S mutation in the mice during the course of the experiment of nine days.

### Effect of *E. coli* Nissle and *E. coli* Nissle::*lux*S in the DSS mouse model of acute colitis

**Figure 4:**
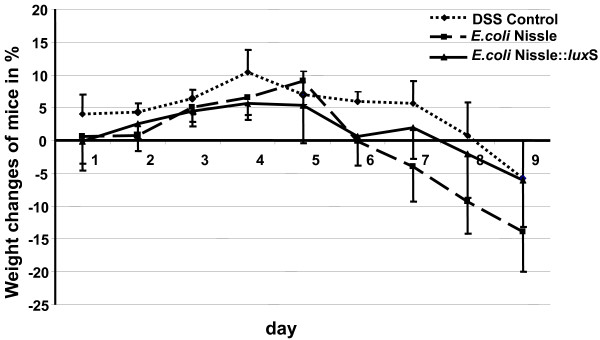
** Changes in body weight in the course of the experiment.** The body weight of each mouse was determined every day during the course of each experiment. “0%” was the weight of the mice, when we started the experiment, the gain/loss of the weight was determined as a percentage value.

The change in weight is a good and easy way to monitor the health status of mice. The development of colitis will lead to weight loss due to the inability to absorb nutrients in the intestine. Thus, the weight of each mouse was measured every day during the course of each experiment. Interestingly, the weight changes of the mice in the different groups were different. Surprisingly, the mice being fed with the wild type *E. coli* Nissle lost more weight on average than the mice being fed with the *E. coli* Nissle::*lux*S or the mice which received 4% DSS in their drinking water only. At the end of each experiment the average weight loss of the mice receiving the *E. coli* Nissle::*lux*S mutant or only 4% DSS was similar. While their weight loss was 5%, the average weight loss of the mice being fed with *E. coli* Nissle was 15% (Figure 4). In respect to the fur and how the mice move, there was a significant difference between the groups of mice. The group infected with the wild type *E. coli* Nissle looked very sick, with their fur being scrubby and their movement around the cage being rather erratic.

**Figure 5:**
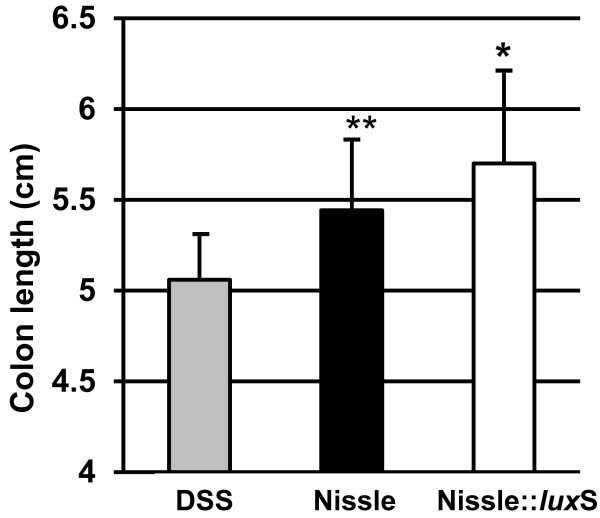
** Length of the colon.** The length of the colon of each mouse was measured, the average colon lenght was determined and graphically displayed. * p<0.05 compared to DSS control; ** p<0.1 compared to DSS control.

The colon length is another valuable measurement to determine the severity of a colitis. During the course of colitis, the colon is shortened. Compared to the DSS control, the length of the colon from the mice being infected with the *E. coli* Nissle::*lux*S was significant longer (p<0.05). The colon of the mice infected with the wild type *E. coli* Nissle was longer than the DSS control (moderate significance p<0.1) (Figure 5).

### Expression of cytokines and mBD-1 in the DSS mice

**Figure 6:**
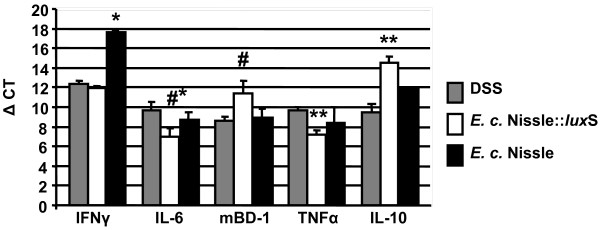
** Expression of cytokines and mBD-1 in the colon.** After scraping the mucosa from the colon, RNA was isolated, transcribed and a semiquantitative RT PCR was run using the primers despicted in Table 1. The ΔCT values were determined and graphically displayed. * p<0.05 compared to *E. coli* Nissle::*lux*S and DSS control; ** p<0.05 compared to DSS control; # p<0.1 compared to Nissle; #* p<0.1 compared to DSS control.

It has been shown by a number of authors, that *E. coli* Nissle is influencing the expression of cytokines and defensins [17,30]. Here we elucidated, if AI-2 is influencing their expression. We have chosen a number of cytokines, which are well characterized, both pro- and anti-inflammatory, and also the mouse defensine mBD-1 [31,32] for analysis. After oral infection of the mice with *E. coli* Nissle or its correspondent *E. coli* Nissle::*lux*S mutant, an acute colitis was induced by DSS. Expression of the pro-inflammatory cytokine Interferon-γ was suppressed 32–64 fold (2^5^-2^6^) in mice infected with the *E. coli* Nissle wild type in comparison to the mice being infected with *E. coli* Nissle::*lux*S or not being infected at all. Significant suppression of the anti-inflammatory cytokine IL-10 in mice being infected by the mutant in comparison to the uninfected mice was seen. The suppression was, however not significant in comparison to the mice been fed with the wild type. In the respect to the pro inflammatory cytokines IL-6 and TNF-α an increased expression of about 8 fold was seen in mice infected with the mutant in comparison with the DSS control (moderate significance of p<0,1) and the wild type. The expression of the mBD-1 was suppressed about 4 fold in mice being infected with the *lux*S mutant in comparison to the wild type infected mice (moderate significance of p<0.1) (Figure 6). We also measured the expression of cytokines in mice which were not treated with DSS and bacteria. In these mice the expression of cytokines was suppressed (data not shown). 

### Histologic examination of the colon from the DSS mice

**Figure 7:**
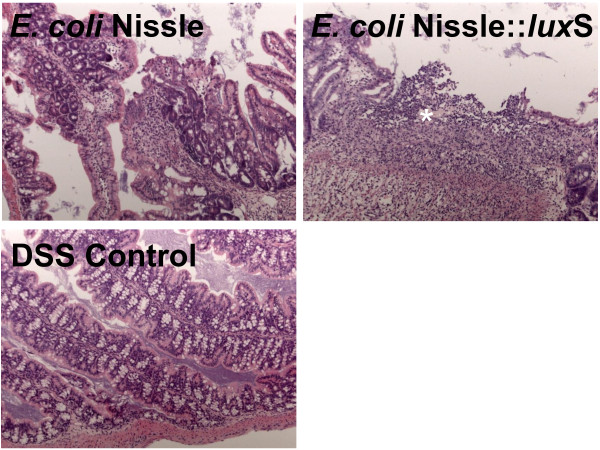
** Histology of the colon.** The paraffin embedded sections of the colon were stained with H & E and were viewed in a blinded fashion by a single pathologist. Patchy inflammation is marked by a star.

The histology of the colon was examined by a single pathologist in a blinded fashion. A scoring system was used to evaluate the inflammation. Regions of patchy inflammations were seen, as denoted by infiltration of granulocytes (see star). Taken together, no significant differences in the pathology between the different groups of mice were seen (Figure 7).

## Discussion

*E. coli* Nissle 1917 is a well established probiotic bacterium. Since its first isolation and description 95 years ago, the interest in this bacterium has increased steadily. Especially during the last few years, the understanding on the mechanisms this bacterium is employing for its beneficial effects, has increased substantially [23]. However, the exact mechanism of action still remains to be elucidated to completely “understand” this bacterium. One important gene regulation mechanism, QS, has, so far, not been looked at in *E. coli* Nissle. It is a density dependent genetic regulatory mechanism which allows single bacterial cells to measure the concentration of certain bacterial signal molecules. In case that *E. coli* Nissle is using QS, we would like to study its influence on the probiotic properties of the bacterium.

Not surprisingly, this bacterium, as all other *E. coli* strains having been studied so far, is not synthesizing homoserine lactones (AI-1 molecules) [13]. However, we showed for the first time, that AI-2 is produced by *E. coli* Nissle in a density dependent manner. During the logarithmic and late logarithmic growth phase the amount of AI-2 being produced is the largest. This finding suggests, that *E. coli* Nissle is using interspecies communication and is “talking” with other bacterial species. In order to study the effect of AI-2 in *E. coli* Nissle, we silenced the corresponding gene via intron insertion. We performed control experiments and were able to show, that no AI-2 was produced by the mutant any longer.

We studied the effect of *lux*S silencing of *E. coli* Nissle in the DSS mouse model of acute colitis. The wild type *E. coli* Nissle and the *E. coli* Nissle::*lux*S mutant behaved similar in respect to colonisation and stability, which is essential in animal experiments. Thus, AI-2 in *E. coli* Nissle is not necessary for survival of the bacterium, otherwise we would not have been able to isolate the mutant bacterium after days in the mouse intestine. Measuring the body weight of the mice during the course of the experiments showed that the mice which were fed with the wild type *E. coli* Nissle lost 15% of their initial weight. The other two groups of mice (*E. coli* Nissle::*lux*S; DSS control) lost on average only 5% of their weight. In addition, the group of mice fed with the *E. coli* Nissle started to lose weight about two days early than the other two groups. In accordance with these data, these mice looked sicker: their fur was scrubby and their movement around the cages was rather erratic. We can speculate, that *E. coli* Nissle is fitter than its mutant, which makes the bacterium more prone for potential translocation: while under healthy conditions there is minimal translocation of intestinal bacteria in mesenterial lymph nodes, under inflammatory conditions the epithelial barrier is broken down and intestinal bacteria and also *E. coli* Nissle are translocated through the Peyer patches and the MLN [33]. The length of the colon is proportional to the level of its inflammation: increasing levels of inflammation results in shortening of the colon. The colon of these mice, which received only DSS, were the shortest, while the colon of the mice, which were inoculated with *E. coli* Nissle or its corresponding mutant were longer. To elucidate the effect of the oral infection of mice with *E. coli* Nissle wild type or its corresponding *lux*S mutant on the expression of cytokines, we choose four well characterized pro- and anti-inflammatory cytokines and one defensine. In mice, which were fed with the wild type *E. coli* Nissle, the pro-inflammatory cytokine IFN-γ, was suppressed in the colon mucosa, while the anti-inflammatory cytokine IL-10 was suppressed by *E. coli* Nissle::*lux*S. The expression of the pro-inflammatory cytokines Il-6 and TNF-α was around 8 fold higher in mice infected with the *lux*S mutant, than in mice of the other two groups. On the other hand the expression of mBD-1 was suppressed in the mice infected with the mutant, in comparison to the mice which were infected with *E. coli* Nissle or were not infected at all. Taken together, we observed significant differences in the expression of cytokines between the different groups of mice. Eventhough the results were partly only moderately significant (p<0.1), we have generated evidence that AI-2 from *E. coli* Nissle is indeed influencing the expression of cytokines and defensins and thus may influence the probiotic properties. It is well known that *E. coli* Nissle is capable of inducing anti-inflammatory cytokines. Early on it was shown, that *E. coli* Nissle is stimulating the epithelial defense in Caco-2 cells [17]. The same authors showed that patients with Crohns disease, who have a mutation in NOD2 have a low level of expression of the defensins HD-5 and HD-6 [17]. The normal colon mucosa is producing human beta-defensin-1 (hBD-1; the homolog to mBD-1) [34]. The functional importance of defensins was shown in elegant experiments with HD-5 expressing transgenic mice. These mice became resistant towards an infection with salmonellae [35]. While pathogenic bacteria seem to suppress the production of defensins probably for self-defense [36], *E. coli* Nissle is protecting its host by defensin induction [17,34].

It has been shown in a number of studies that homoserine lactones (AI-1) regulate the expression of cytokines and virulence factors, for example in *Vibrio cholerae*, *Pseudomonas aeruginosa* or EHEC [37-39]. However, the influence of AI-2 on the cytokine expression was discussed only in two reports so far [40,41]. Using microarrays the group showed the differential regulation of a number of genes involved in the complement pathway, regulation of cytokine expression and antigen presentation when infecting RAW264.7 macrophages with the wild type *Vibrio vulnificus* or with the corresponding *lux*S mutant.

Is QS also used by other probiotic bacteria? Studies were published only with bacteria of the genus *Lactobacillus*. Early on it was shown that *Lactobacillus rhamnosum* GG is communicating via AI-2 molecules [42]. It was shown that the *lux*S gene has a clear role in the acidic stress response; AI-2 activity increased by lowering the pH in a dose dependent manner [43]. A second probiotic bacterium, *L. acidophilum* strain La-5, showed that its supernatant is influencing the AI-2 concentration and the expression of virulence genes of the enterohemorrhagic *E. coli* (EHEC) 0157:H17 in the gut, inhibiting its colonization. In addition it was shown, that the supernatant is reducing the attaching and effacing lesions in HeLa cells. Also a significant inhibition of bacterial adhesion in Hep-2 cells was observed. In elegantly designed mouse experiments, the authors used slow-scan CCD cameras to show reduced fecal shedding of luminescent EHEC constructs when *L. acidophilus* was fed additionally [44,45]. Another *Lactobacillus* species, *L. plantarum* was used successfully to inhibit the pathogenic activity of *Pseudomonas aeruginosa*. The AHL production, as well as the production of elastase and biofilm was inhibited by *L plantarum* cultures and filtrates, but not by isolated, washed cells [46]. In another set of experiments with *L. plantarum*, it was shown that its LamBDCA quorum sensing system is responsible for the modulation of cytokine response in human PBMC [47]. These studies showed that indeed QS is influencing the probiotic properties of the studied *Lactobacillus* species. In the future, QS in other probiotic species should be studied, to hopefully confirm that QS is indeed influencing the probiotic properties.

Examining the histologic sections of the colon of the different groups of mice, we did not observe any significant difference between the groups. In contrast to the histopathology of a chronic inflammation, *E. coli* Nissle has no influence in case of an acute DSS induced colitis. Here one can observe histologic patchy mucosal damage with the loss of crypts, followed by the acute transmural infiltration of inflammatory cells. No T- or B-cells are necessary [48].

In this report we showed for the first time that the probiotic bacterium *E. coli* Nissle is producing AI-2 molecules. Here we show that AI-2 is affecting the regulation of cytokine expression in the DSS mouse model of acute colitis. In comparison to the *E. coli* Nissle *lux*S mutant, the mice which were infected with the wild type lost more weight, looked sicker regarding their fur and their lack of movement. On the other hand, mice, which were infected with the *E. coli* Nissle::*lux*S mutant showed a higher expression of pro inflammatory cytokines, but a reduced expression of the anti-inflammatory cytokine IL-10 or the mBD-1. Thus, it remains to be seen if AI-2 is influencing the probiotic properties of this important bacterium.

## Competing interest

The authors declare that they have no competing interests.

## Authors’contribution

CAJ designed and performed most of the experiments. He supervised the experiments which were not performed by him. He has written the manuscript. The work of SG focused on the in vitro experiments while C-JH performed parts of the RT PCR and gave valuable advice. JSF introduced CAJ into the DSS mouse model and was of great help concerning the mouse experiments. PA was examining the histological sections. GL, IBA, MG and PM gave valuable advice concerning the experiments and for the preparation of the manuscript. All authors read and approved the manuscript.

## Supplementary Material

Additional file 1 ** Construction of the *E.coli* Nissle::*lux*S mutant; including control experiments: A: Schematic diagram of the construction of the mutant: left: *E.coli* Nissle wild type, right: *E.coli* Nissle::*lux*S mutant.** B: control PCR: Intron is inserted into the *lux*S gen (the different colored arrows are symbolizing different primer pairs) M: Marker; numbers are in kilobases. C: *E.coli* Nissle wild type (WT) produces AI-2 while the *E.coli* Nissle::*lux*S mutant does not. Click here for file
