# Supplementary figures and images for: Quorum sensing in the probiotic bacterium Escherichia coli Nissle 1917 (Mutaflor) – evidence that furanosyl borate diester (AI-2) is influencing the cytokine expression in the DSS colitis mouse model
Source: Gut Pathog. 2012 Aug 3;4:8. doi: 10.1186/1757-4749-4-8 (PMC3480846; doi:10.1186/1757-4749-4-8)

## Slide 1
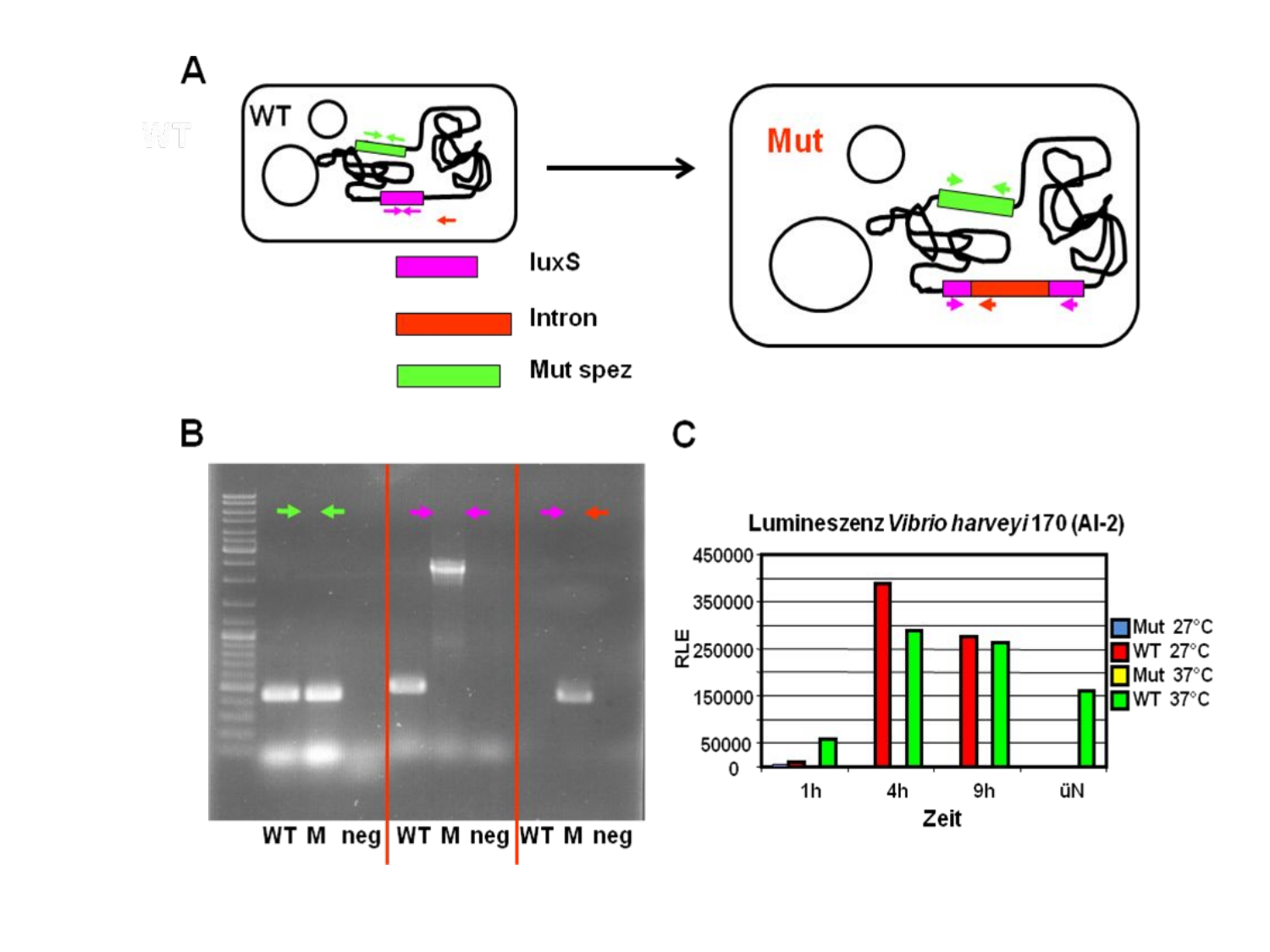

Supplement: Additional file 1 — Construction of the E.coli Nissle::luxS mutant; including control experiments: A: Schematic diagram of the construction of the mutant: left: E.coli Nissle wild type, right: E.coli Nissle::luxS mutant. B: control PCR: Intron is inserted into the luxS gen (the different colored arrows are symbolizing different primer pairs) M: Marker; numbers are in kilobases. C: E.coli Nissle wild type (WT) produces AI-2 while the E.coli Nissle::luxS mutant does not. [file 1757-4749-4-8-S1.ppt]
